# Transcriptome sequencing of adenomyosis eutopic endometrium: A new insight into its pathophysiology

**DOI:** 10.1111/jcmm.14718

**Published:** 2019-10-01

**Authors:** Yuqian Xiang, Yabing Sun, Bingxin Yang, Yeping Yang, Ying Zhang, Tiantian Yu, Hefeng Huang, Junyu Zhang, Hong Xu

**Affiliations:** ^1^ International Peace Maternity and Child Health Hospital School of Medicine, Shanghai Jiao Tong University Shanghai China; ^2^ Shanghai Key Laboratory of Embryo Origianl Diseases Shanghai China; ^3^ Shanghai Municipal Key Clinical Specialty Shanghai China

**Keywords:** adenomyosis, C/EBPβ, DNA methylation, eutopic endometrium, RNA sequencing

## Abstract

The eutopic endometrium has been suggested to play a crucial role in the pathogenesis of adenomyosis. However, the specific genes in eutopic endometrium responsible for the pathogenesis of adenomyosis still remain to be elucidated. We aim to identify differentially expressed genes (DEGs) and molecular pathways/networks in eutopic endometrium from adenomyosis patients and provide a new insight into disease mechanisms at transcriptome level. RNA sequencing (RNA‐Seq) was performed with 12 eutopic endometrium from adenomyosis and control groups. Differentially expressed genes in adenomyosis were validated by quantitative real‐time PCR (qPCR) and immunochemistry. Functional annotations of the DEGs were analysed with Ingenuity Pathway Analysis (IPA). Quantitative DNA methylation analysis of *CEBPB* was performed with MassArray system. A total of 373 differentially expressed genes were identified in the adenomyosis eutopic endometrium compared to matched controls. Bioinformatic analysis predicted that IL‐6 signalling and ERK/MAPK signalling were activated in adenomyosis endometrium. We also found that the increased expression and DNA hypomethylation of *CEBPB* were associated with adenomyosis. Our results revealed key pathways and networks in eutopic endometrium of adenomyosis. The study is the first to propose the association between C/EBPβ and adenomyosis and can improve the understanding of the pathogenesis of adenomyosis.

## 1 INTRODUCTION

Adenomyosis is a common and chronic gynaecological disease that affects 10%‐80% of women of reproductive age worldwide.^1^, ^2^ It is characterized by the presence of endometrial stroma and glands within the myometrium and its main clinical features include chronic pelvic pain, abnormal uterine bleeding, menorrhagia, dysmenorrhea, dyspareunia and infertility.^1^, ^3^, ^4^, ^5^ In recent years, due to the advancement of imaging techniques such as transvaginal ultrasound scan (TVUS) and magnetic resonance imaging (MRI), substantial evidences suggest that adenomyosis may develop more often among younger women.^6^, ^7^ To date, no novel compound is reported to develop for adenomyosis and clinical studies on the well‐conducted pharmacologic treatment are seldom reported.^8^ The most effective treeatments of adenomyosis are invasive surgeries such as hysterectomy, which is unacceptable for the patients who want to preserve their fertility. As a result, it is necessary to explore the molecular mechanisms underlying the disease and develop adequate therapeutic strategies.

The precise aetiology and physiopathology of adenomyosis are far from being completely understood and several theories including sex steroid hormone aberrations, altered cell proliferation, inflammation and neuroangiogenesis have been established to interpret the manifestations of the disease. According to the most widely accepted theory, adenomyosis derives from the down‐growth and infiltration of endometrial basal layer deep into the myometrium through an absent or altered junctional zone (JZ), indicating the important role of the eutopic endometrium.^9^, ^10^

To date, most studies on adenomyosis are limited to the evaluation of expression alterations of one or several particular genes, but the systematic studies on the differentially expressed genes or the primary pathways implicated in the initiation of adenomyosis are seldom reported. The transcriptome interpretation is required for understanding the development and pathogenesis of the disease. There is one and only report about the global transcriptome abnormalities of the eutopic endometrium in proliferative stage from women with adenomyosis by Herndon et al,^11^ in which they compared the transcriptome between three cases and five controls with the Gene 1.0 ST Affymetrix platform and identified many abnormally expressed genes, pathways and networks. RNA sequencing (RNA‐Seq) is well‐established approach to decipher the entire transcriptome using next‐generation sequencing in a high‐throughput and quantitative manner.^12^ Our study aims to identify differentially expressed genes (DEGs) and molecular pathways/networks in eutopic endometrium from the patients with adenomyosis and provide new insights into disease mechanisms at transcriptome level with RNA‐Seq.

## 2 MATERIALS AND METHODS

### 2.1 Patients and clinical samples

Adenomyosis patients were diagnosed and recruited according to typical clinical symptoms of pelvic pain, abnormal uterine bleeding and dysmenorrhea, physical examination results, and imaging reports including transvaginal ultrasound and MRI reports. All samples were collected from the International Peace Maternity & Child Health Hospital (IPMCH), Shanghai Jiao Tong University School of Medicine. All participants enrolled in our present study were Han Chinese, Asia population.

Thirty patients with adenomyosis and 31 women with no adenomyosis as matched controls were enrolled in our study. All participants enrolled in our study were premenopausal. The recruited patients had regular menstrual cycles (26‐32 days), and the participants had not received hormonal treatments or used intrauterine contraception for at least 6 months prior to surgery. All endometrium samples in our manuscript were collected through curettage during surgery, and they were the functionalis. For segmental adenomyosis, the eutopic endometrium samples were collected during hysterectomy or adenomyomectomy according to their surgery options. For diffuse adenomyosis, the eutopic endometrium samples were collected during hysterectomy. The endometrium from controls was also collected through uterine curettage from women with non‐endometrial pathogenesis such as myoma of uterus and was laparoscopically confirmed to be free of adenomyosis or endometriosis. As for collecting ectopic endometrial tissue samples, the whole piece of adenomyosis lesion were removed from the uterus during hysterectomy or adenomyomectomy. The collected lesions were immediately sent to be histologically confirmed adenomyosis according to the presence of stroma and endometrial glands at least one lower‐power field of view away from the endometrial‐myometrial junction. After taking adenomyosis lesions away from operating table, we dissected it into small pieces with surgical scissors and saved them as three parts. Samples were immediately fixed in 10% buffered formalin and then processed for paraffin embedding as the first part, maintained in centrifuge tubes with RNAlater (Ambion Inc) and stored at −80°C as the second part, preserved in cryotube and stored at −80°C as the third part.

The study protocols were approved by the Ethics Review Committee of IPMCH and conducted according to the Declaration of Helsinki Principles. Written informed consents were obtained from all the participants of this study.

### 2.2 RNA sequencing (RNA‐Seq) and data analysis

Given the cyclical changes in endometrium, the eutopic endometrium of subjects used for RNA‐Seq analysis was all in the proliferative phase of the menstrual cycle, as confirmed by endometrial histology. Total RNA was isolated with RNeasy mini‐kit, followed by RNase‐Free DNase treatment (QIAGEN) according to the manufacturer's instructions. RNA‐Seq library preparation had been described in detail.^13^ Briefly, the Illumina TruSeq RNA Library Prep Kit (Illumina) was used for the preparation of mRNA‐seq library. The quality and concentration of all libraries were analysed with an Agilent Bioanalyzer (Agilent). mRNA sequencing was performed on Illumina Hiseq 2500 sequencing system (Illumina), and 150‐bp paired‐end FASTQ read files were generated.

Raw mRNA‐seq data were trimmed using Trimmomatic v.0.33.^14^ Clean reads were aligned with the UCSC *Homo sapiens* reference genome (build hg19) using TopHat v.2.1.0,^15^ followed by transcript assembly and differential transcripts expression analysis using Cufflinks v.2.2.1.^15^ The coverage rates of transcripts were obtained by Fragments Per Kilobase per Million (FPKM), which was calculated based on the length of the transcript and the number of reads mapped to this gene/transcript. mRNAs with absolute value Log_2_Ratio ≥ 1 and *q*‐values < .05 were marked as significant.

### 2.3 cDNA preparation and quantitative PCR

Total RNA was isolated with the TRIzol reagent (Thermo Fisher Scientific) according to the manufacturer's protocol. The RNA concentration and purity were evaluated with NanoDrop ND‐2000 spectrophotometer (Thermo Fisher Scientific Inc). With 1 μg of RNA in the 20 μL reaction system, reverse transcription reactions were performed with PrimeScript Reverse Transcriptase (Takara Bio) according to the manufacturer's instructions.

To validate the confidence of RNA‐Seq, several differentially expressed genes were selected and analysed by quantitative real‐time PCR (qPCR) utilizing the SYBR Premix EX Taq reagent (Takara) in a QuantStudio 7 Flex Real‐Time PCR System (Applied Biosystems). Primers (Table S2) were designed for the coding sequences of the candidate genes in Primer 3 software (http://frodo.wi.mit.edu/cgi-bin/primer3). *GAPDH* was used as the internal control. qPCR replicates were performed in a final volume of 10 μL containing primers, SYBR Premix EX Taq reagent (Takara) and cDNA templates. The relative expression levels of the candidate genes were calculated as the averaged normalized Ct value of each sample compared with the *GAPDH* Ct value of the corresponding sample based on the 2^−ΔΔCt^ method.

### 2.4 Functional analysis and network generation

Top biological functions and canonical pathways associated with the differentially expressed mRNAs data set were identified with Ingenuity Pathway Analysis (IPA) (Qiagen).^16^ Fisher's exact test was performed to determine the probability that each biological function or canonical pathway assigned to the data set could be explained by chance alone. Molecular interaction networks were algorithmically generated based on the molecule connectivity. Network scores were calculated with Fisher's exact test and were equal to the −log_10_ (*P*‐value). Downstream effect analysis was used to predict downstream biological processes and infer their activation states based on the observed gene expression changes in the data set. A *z*‐score was calculated to infer the activation states (‘increased’ or ‘decreased’) of implicated biological processes.^17^

### 2.5 Immunohistochemistry

Fresh tissues were collected and fixed in 4% paraformaldehyde for 24 hours and then embedded in paraffin. The embedded tissues were cut into 4 μm thick sections and mounted onto glass slides. After deparaffinization, dehydration, rehydartion and antigen retrieval, H_2_O_2_ and BSA were used to deactivate the endogenous peroxidase and block the nonspecific binding, respectively. The above treated tissue sections were incubated with primary antibody overnight at 4°C, followed by the incubation with secondary antibody. The staining was performed with DAB and haematoxylin. The glass slides were mounted in Permount™ Mounting Medium. Six random views were captured by microscope (Leica, DM2000). The data were analysed by Image‐pro plus 6.0 to measure the value of IOD.

### 2.6 Quantitative DNA methylation analysis using MassARRAY and transcription factor binding site prediction

Quantitative DNA methylation analysis was performed with MassARRAY system (Sequenom) as described previously.^18^ Briefly, genomic DNA was isolated, followed by bisulphite conversion. After amplification and MassCLEAVE base specific cleavage, the PCR products were analysed by mass spectrometry. The spectra and methylation values were collected and analysed using EpiTYPER software. The primers for the *CEBPB* gene were designed with the online software Epidesigner (http://www.epidesigner.com) (Table S2). The analysed amplicon represents a 544‐bp fragment (positions −588 bp to −45 bp) in the vicinity of transcriptional start site (TSS) of *CEBPB* gene. The bioinformatic tool LASAGNA‐Search 2.0^19^ was used to search for transcription factor binding sites within the analysed *CEBPB* amplicon.

### 2.7 Statistical analysis

Clinical and experimental data were expressed as mean ± SD and analysed through two‐tailed Student's *t* test or Fisher exact test based on the type of data in the IBM SPSS Statistics 24 (IBM Corporation). Hierarchical cluster analysis clustered the expression of DEGs based on Euclidean distance and the complete linkage clustering algorithm. Fisher's exact test was used to identify pathways and networks with the statistical significance correlated with DEGs identified in the study. The IPA *z*‐score algorithm was used to predict the activation state for a given biological function. A *z*‐score ≥ 2 means a significantly increased function, whereas a *z*‐score ≤ 2 indicates that a function is significantly decreased. For all analyses, *P* < .05 was considered to be statistically significant.

## 3 RESULTS

### 3.1 Clinical characteristics in the adenomyosis and non‐adenomyosis groups

The 30 patients with adenomyosis enrolled in our study included one nonparous and 29 multiparas who were aged from 30 to 53 with a mean age of 44.53 ± 5.61. Both eutopic and ectopic endometrium were collected. Eighteen patients were in the proliferative phase, and 12 patients were in the secretory phase. Among the 30 enrolled women with adenomyosis, 25 patients suffered from dysmenorrhea. Considering the kinds of adenomyosis, 20 patients were diffuse, and another 10 patients were segmental. The 31 matched controls were all multiparas who aged from 30 to 54 with a mean age of 46.74 ± 4.69. The endometrium was collected when matched controls underwent hysterectomy. Among the match controls, 16 tissues were in the proliferative phase, and 15 tissues were in the secretory phase. None of the matched controls suffered from dysmenorrhea. The clinical details of the participants are provided in Table S1.

### 3.2 RNA‐Seq analysis and identification of differentially expressed genes

**Figure 1:**
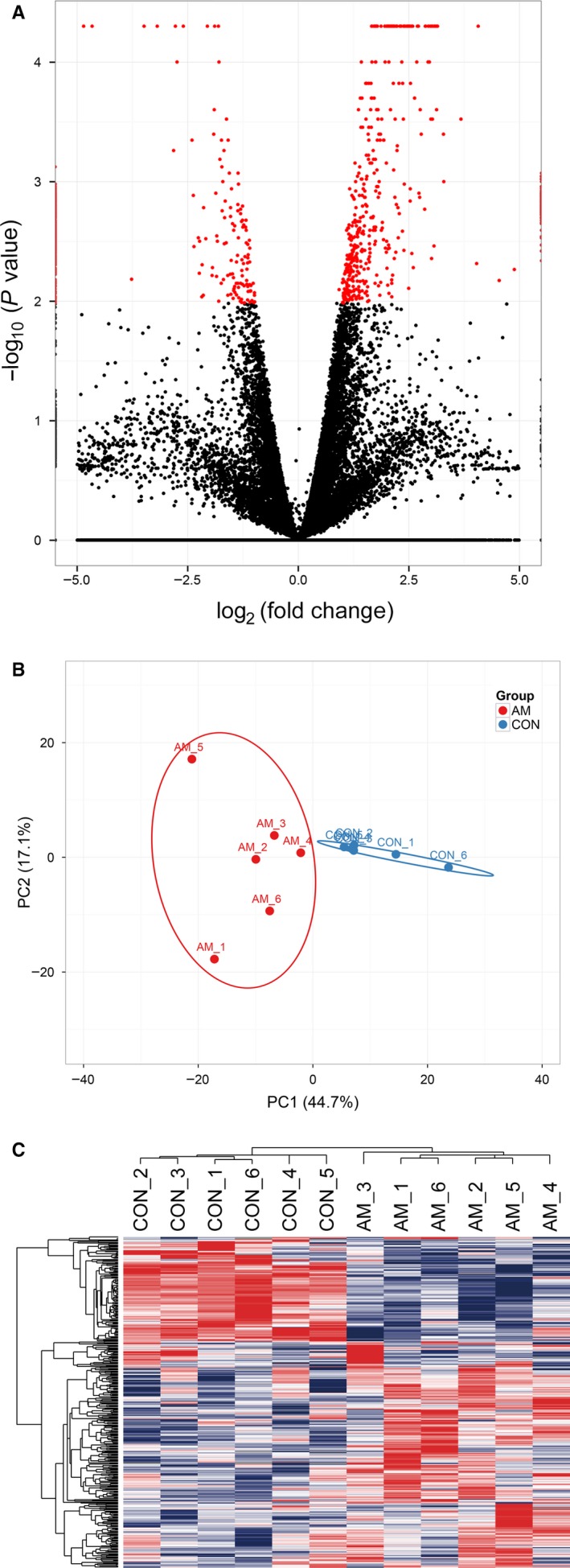
Differentially expressed genes between endometrium from women with adenomyosis and matched controls identified by RNA‐Seq. A, Volcano plots of genes with differential expression. The *x* axis represents the log_2_ (fold change), and the *y* axis represents −log_10_(*P* value) calculated by student's *t* test. The red points represent the identified genes with statistical significance (*P* < .05 and fold change ≥ 2). B, Principal component analysis (PCA). C, Hierarchical clustering analysis of genes with differential expression

To better understand the pathogenesis of adenomyosis, we conducted a comparative transcriptomic analysis of eutopic endometrium between six patients with adenomyosis and six women without adenomyosis. We obtained about an average of 28 million read pairs from each sample and further compared the expression profiles in eutopic endometrium between adenomyosis and control groups. Totally, 22 836 annotated mRNAs were identified and 373 mRNAs (*q*‐value < .05; absolute value Log_2_Ratio ≥ 1) were significantly deregulated (Figure 1A). Among the identified differentially expressed genes mentioned above, 258 genes were up‐regulated and 115 genes were down‐regulated in eutopic endometrium from patients with adenomyosis compared to that in the control subjects (Table S3). Principle component analysis (PCA) and hierarchical clustering were performed with the data sets of differentially expressed mRNAs. The PCA result illustrated that the eutopic endometrium from adenomyosis exhibited distinct gene expression profiles compared with control endometrium (Figure 1B). Hierarchical clustering analysis of the differentially expressed genes in eutopic endometrium from women with adenomyosis and control group indicated that the gene expression patterns clustered separately after unsupervised clustering (Figure 1C). These results suggested a profound impact of adenomyosis on eutopic endometrial mRNA expression.

### 3.3 Validation of mRNA expression

**Figure 2:**
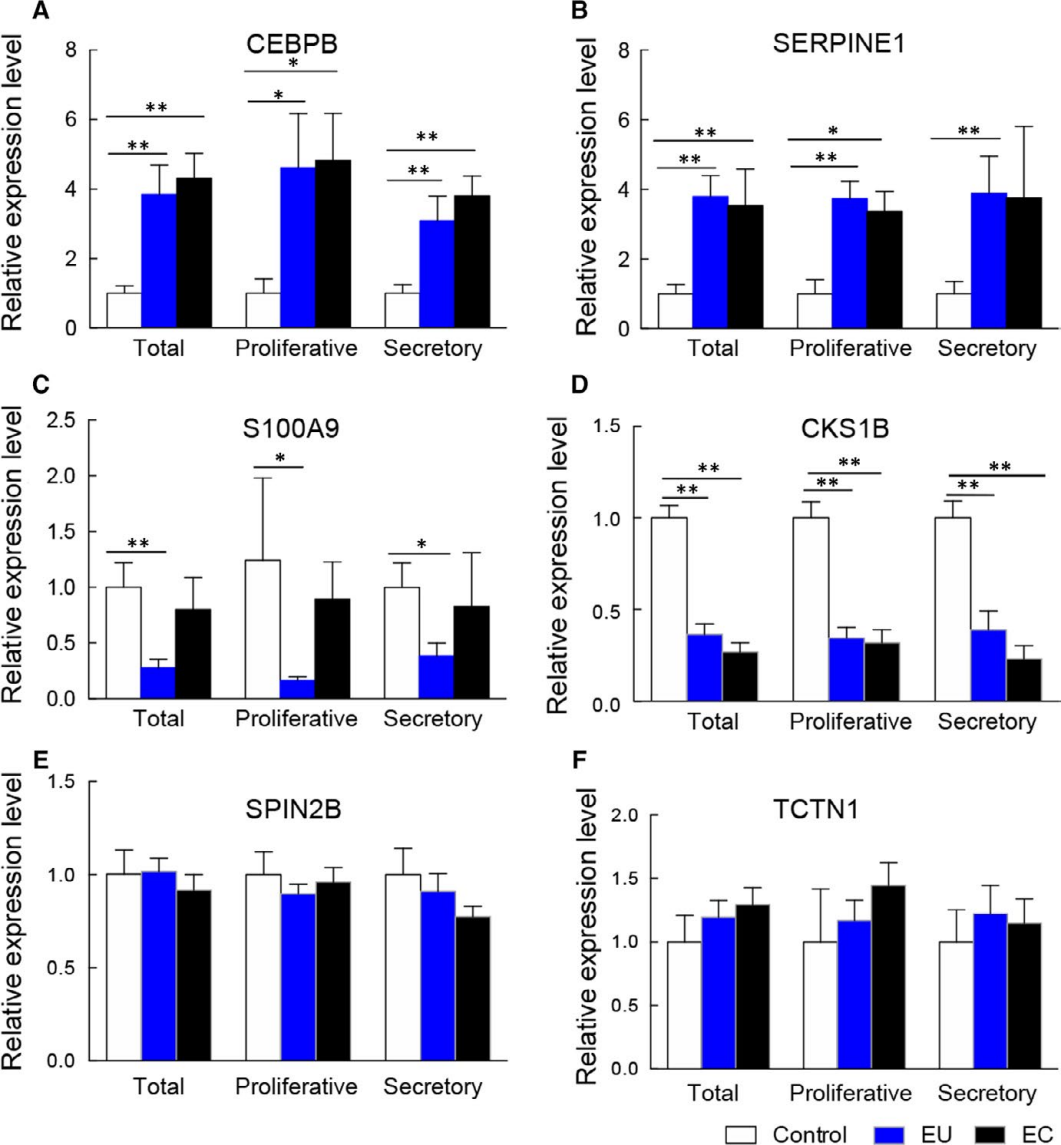
Validation of genes expression by real‐time PCR in eutopic, ectopic endometrium (N = 14, 7 in proliferative stage and 7 in secretory stage) and matched controls (N = 15, 5 in proliferative stage and 10 in secretory stage). A, CEBPB, B, SERPINE1, C, S100A9, D, CKS1B, E, SPIN2B and F, TCTN1. EC, ectopic endometrium; EU, eutopic endometrium; **P < .01, *P < .05

To confirm the accuracy of RNA‐Seq, six genes including up‐regulated (*CEBPB*, *SERPINE1*), down‐regulated (*S100A1*, *CKS1B*) and unchanged (*SPIN2B*, *TCTN1*) between endometrium from women with adenomyosis and controls were selected for further validation in additional set of clinical samples. Fourteen women with adenomyosis including seven in proliferative phase and seven in secretory phase were enrolled in the replication round. Both eutopic and ectopic endometrium of the 14 patients were collected. Other 15 matched endometrium of controls were incorporated for replication, among them, five patients were in the proliferative phase and 10 patients were in the secretory phase. Consistent with our expectation, the validation results were consistent with the RNA‐Seq data (Figure 2). Meanwhile, other genes including *BTG1*, *CD69*, *CYR61*, *DUSP1*, *DUSP5*, *EMP1*, *F3*, *SOCS3* and *WEE1* were also selected for validation, which are shown in Figure S1.

### 3.4 Functional analysis of differentially expressed genes

We performed functional analysis with Ingenuity Pathway Analysis (IPA, Ingenuity Systems) to elucidate biological functions affected by DEGs. Ingenuity Pathway Analysis identified significantly enriched biological processes associated with DEGs (Table 1). From the perspective of diseases and disorders, neurological disease (*P*‐value: 3.41E−06 ‐ 2.57E−30, 67 genes assigned) and organismal injury and abnormalities (*P*‐value: 4.56E−06 ‐ 2.57E−30, 336 genes assigned) were the top two significantly enriched terms. From the perspective of molecular and cellular functions, cellular growth and proliferation (*P*‐value: 4.48E−06 ‐ 2.10E−29, 199 genes assigned) and cellular movement (*P*‐value: 4.48E−06 ‐ 8.36E−29, 142 genes assigned) were the top two significantly enriched terms. From the perspective of physiological system development and functions, tissue morphology (*P*‐value: 4.04E−06 ‐ 4.42E−25, 144 genes assigned) and haematological system development and function (*P*‐value: 4.48E−06 ‐ 9.55E−14, 135 genes assigned) were the top two enriched terms.

**Table 1:** Functional analysis for the differentially expressed genes in endometrium from women with adenomyosis compared with matched controls

Top diseases and bio functions	*P*‐value^a^	# of genes
Diseases and disorders		
Neurological disease	3.41 E−06 ‐ 2.57 E−30	67
Organismal injury and abnormalities	4.56 E−06 ‐ 2.57 E−30	336
Connective tissue disorders	2.97 E−06 ‐ 4.00 E−16	79
Immunological disease	3.40 E−06 ‐ 4.00 E−16	158
Inflammatory disease	3.14 E−06 ‐ 4.00 E−16	97
Molecular and cellular functions		
Cellular growth and proliferation	4.48 E−06 ‐ 2.10 E−29	199
Cellular movement	4.48 E−06 ‐ 8.36 E−29	142
Cell death and survival	4.48 E−06 ‐ 1.45 E−26	177
Cellular development	4.48 E−06 ‐ 1.01 E−21	184
Cellular function and maintenance	4.31 E−06 ‐ 5.79 E−19	164
Physiological system development and function		
Tissue morphology	4.04 E−06 ‐ 4.42 E−25	144
Haematological system development and function	4.48 E−06 ‐ 9.55 E−14	135
Cardiovascular system development and function	4.31 E−06 ‐ 3.63 E−19	110
Organismal development	6.43 E−14 ‐ 5.82 E−19	137
Hematopoiesis	3.54 E−06 ‐ 1.68 E−18	91

a Range of *P*‐value indicates higher level functions that contained multiple lower level functions.

**Figure 3:**
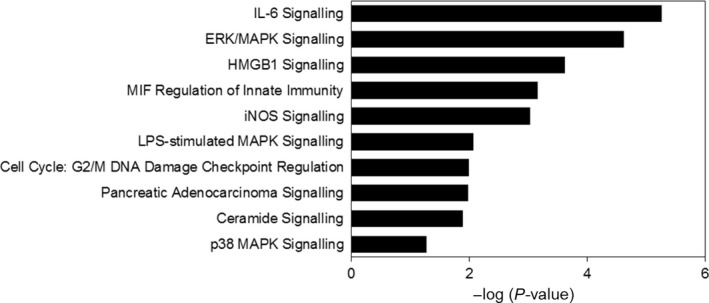
Pathway analysis of genes with differential expression identified by RNA‐Seq

Canonical pathway analysis can give some clues to the biochemical and signal transduction pathways that DEGs may participate in. Figure 3 displayed the top significant canonical pathways associated with DEGs in adenomyosis. ‘IL‐6 signalling’ (−log(*P* value) = 5.87) and ‘ERK/MAPK signalling’ (−log(*P* value) = 5.21) were the major pathways altered in eutopic endometrium of adenomyosis.

**Figure 4:**
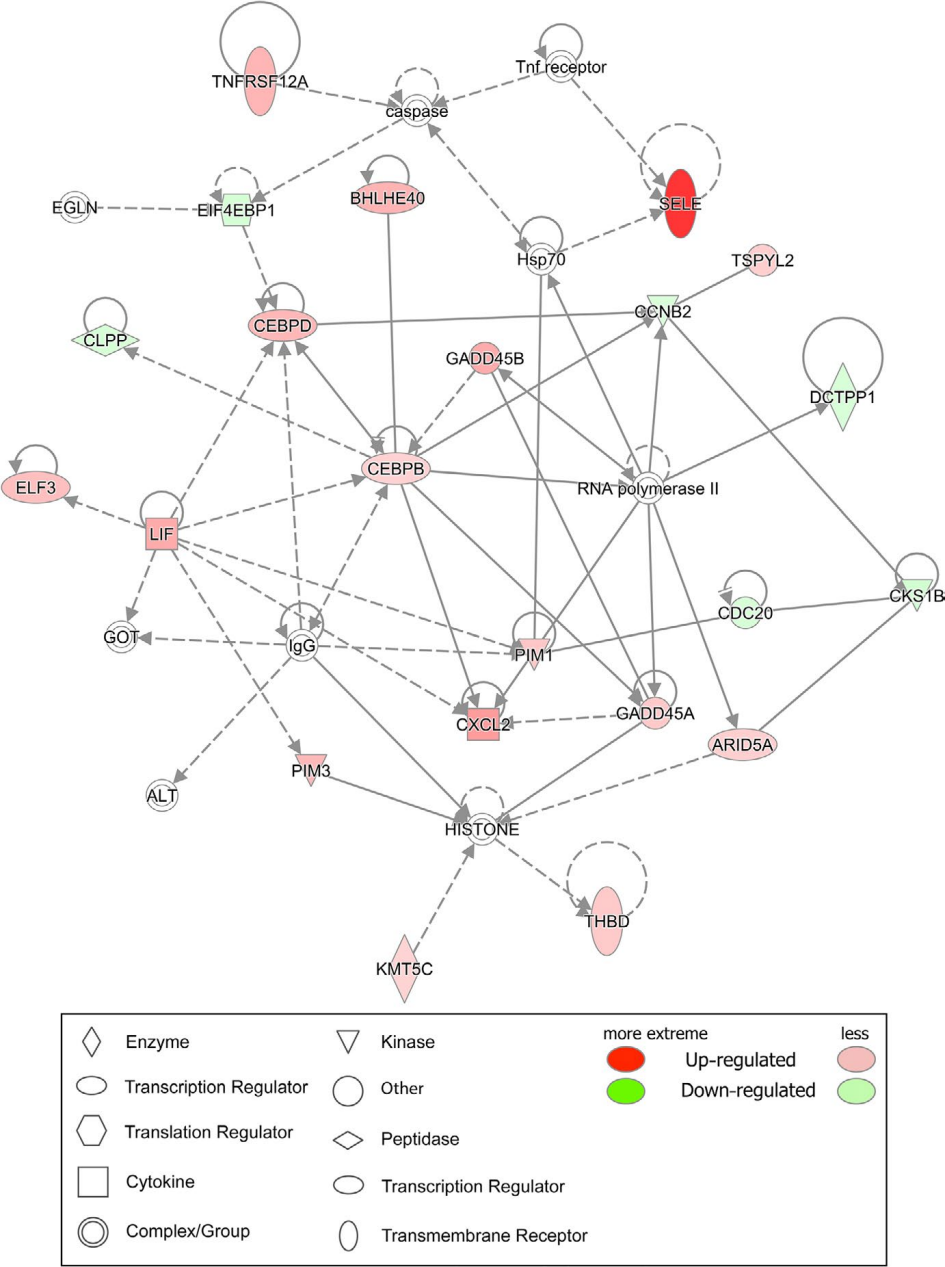
The molecular interaction networks analysis of genes with differential expression. Cellular Development, Cellular Growth and Proliferation, Embryonic Development. Red colour represents that the gene expression level is increased in endometrium from women with adenomyosis, and the darker the colour, the greater in gene expression level. On the opposite, green represents the gene expression level was decreased in endometrium from women with adenomyosis

The molecular interaction networks were further generated to explore the pathogenesis of adenomyosis based on the identified DEGs connectivity and ranked by score. Our results showed ‘Cellular Development, Cellular Growth and Proliferation, Embryonic Development’ (Score = 43) (Figure 4) was the most enriched network in DEGs of adenomyosis. Notably, CCAAT/enhancer‐binding protein β (C/EBPβ, also known as CEBPB) was a central ‘node’ in the network, with the maximum number of nodes.

### 3.5 Immunohistochemical analysis of C/EBPβ

**Figure 5:**
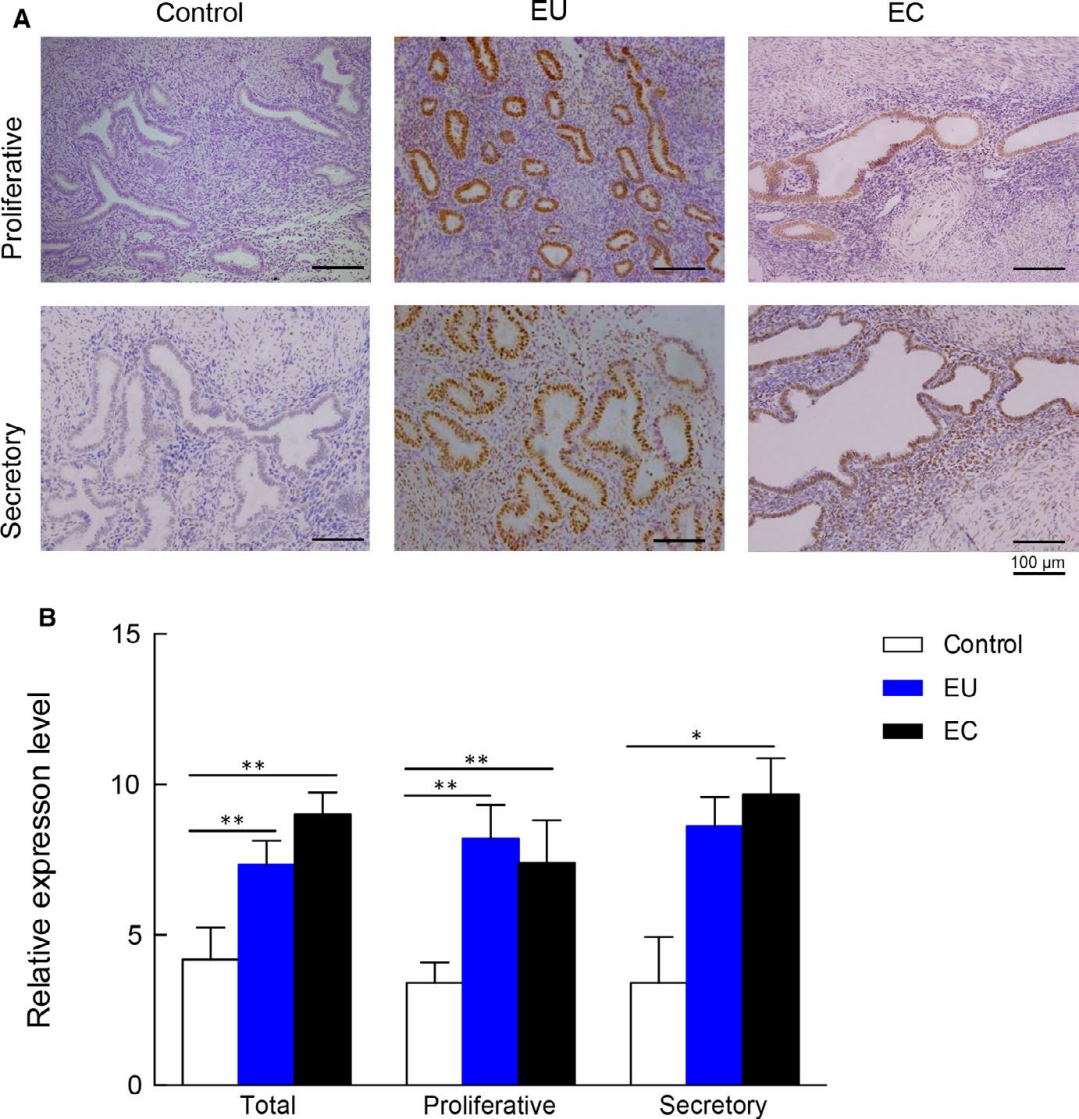
Representative immunohistochemical staining micrograph of C/EBPβ in different endometrial tissues. A, The representative immunohistochemical staining of eutopic, ectopic endometrium and matched controls in proliferative and secretory phase (original magnification 200×). B, The statistical analysis results of immunohistochemical staining from 10 patients with adenomyosis and 10 matched controls. EC, ectopic endometrium; EU, eutopic endometrium; **P < .01, *P < .05

Considering the important roles in the regulation of differentiation and cell survival, we then selected C/EBPβ to further confirm its expression patterns in the endometrium. Immunohistochemical analysis was performed in 10 endometrium of controls and 10 paired eutopic and ectopic endometrium from women with adenomyosis. As a whole, the expression level of C/EBPβ was significantly increased in both eutopic and ectopic endometrium compared with that in matched controls (*P* = .001 and 0.0001, respectively; Figure 5), and the expression level of C/EBPβ in the eutopic endometrium was higher than that in the ectopic endometrium, but the difference was not significant (*P* = .316, Figure 5). Consistent with the whole expression patterns, the immunostaining data showed increased expression of C/EBPβ in both eutopic and ectopic endometrium compared with that in controls during proliferative and secretory phase (Figure 5). Although the expression of C/EBPβ exhibited slightly decreased expression in ectopic endometrium in comparison with that in eutopic endometrium, but with no statistical difference (Figure 5).

### 3.6 Quantitative DNA methylation analysis of *CEBPB*

**Figure 6:**
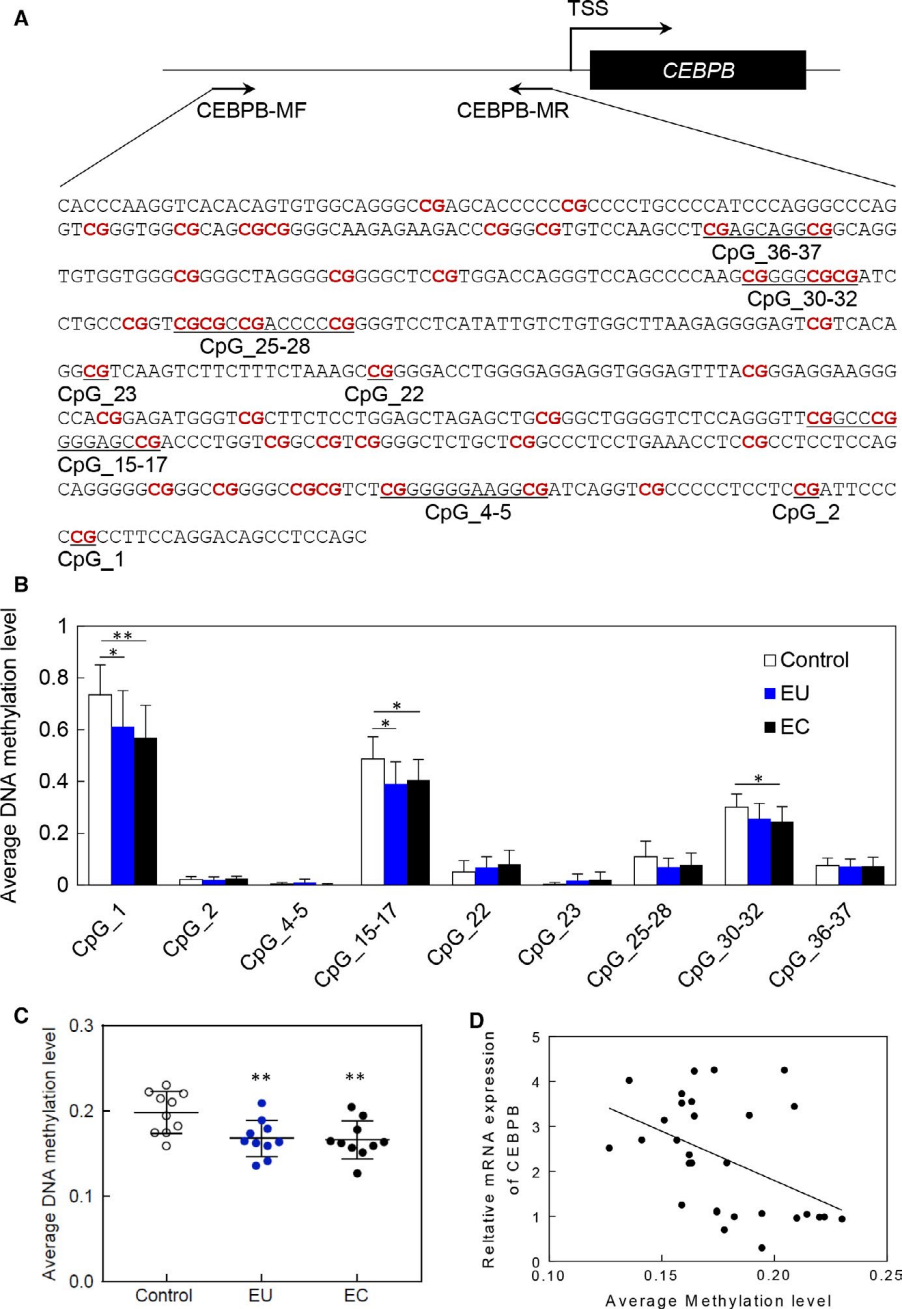
DNA methylation analysis of CEBPB in different endometrial tissues. A, Schematic representation of the TSS and the proximal promoter region of *CEBPB* gene. Positions and orientation of the MassARRAY primers are indicated by black arrows. The analysed CpG sites by MassARRAY are listed in order. B, The average DNA methylation level of each CpG unit in the amplicon among different endometrial tissues. C, The whole DNA methylation level of *CEBPB* among different endometrial tissues. D, Correlation analysis of CEBPB between DNA methylation and gene expression level. EC, ectopic endometrium; EU, eutopic endometrium; **P < .01, *P < .05

DNA methylation plays an important role in the regulation of gene expression. To explore whether the expression of C/EBPβ was influenced by DNA methylation, we further investigated DNA methylation of the *CEBPB* gene. An amplicon including 18 CpG sites (nine units) in CEBPB (−588 bp to −45 bp, relative to transcription start site) was analysed in 10 normal endometrium and 10 paired eutopic and ectopic endometrium from women with adenomyosis using the MassARRAY system (Sequenom) (Figure 6A).

Quantitative DNA methylation analysis showed that the average methylation level of the *CEBPB* amplicon was lower in endometrium from women with adenomyosis, in which the methylation level was 0.168 (*P* = .0084) and 0.166 (*P* = .0064) in eutopic and ectopic endometrium, respectively (Figure 6B,C), and the corresponding methylation level was 0.198 in endometrium from matched controls. Three CpG unites including CpG_1, CpG_15‐17 and CpG_30‐32 showed significantly decreased DNA methylation level in eutopic and ectopic endometrium compared with that in controls, and the mean methylation levels of CpG_1 = 0.611, 0.570 and 0.735 in eutopic, ectopic endometrium and control (*P* = .0449 and 0.0065); the mean methylation of CpG_15‐17 = 0.390, 0.406 and 0.487 in eutopic, ectopic endometrium and control (*P* = .0213 and 0.0411); and the mean methylation level of CpG_30‐32 = 0.245 and 0.301 in ectopic endometrium and controls(*P* = .0330). We further searched the potential role of above 3 CpG units in gene expression using LASAGNA‐Search 2.0,^14^ and the results indicated that CpG_1, CpG_15‐17 and CpG_30‐32 might be the binding sites of GATA‐1 (*P* = .00045), MZF1 (*P* = .000175) and GATA‐3 (*P* = .001), respectively. Correlation analysis showed that the DNA methylation level was negatively correlated to the gene expression of *CEBPB* (Figure 6; *R* = −0.463, *P* = .01) (Figure 6D).

## 4 DISCUSSION

Adenomyosis is a common gynaecological disorder characterized by the presence of endometrial stroma and glands invading the myometrium. Although it is generally accepted that adenomyosis is a distinct disease from endometriosis requiring different therapeutic approaches, the underlying molecular mechanism of adenomyosis remains unclear. To our knowledge, the study is the first time to assess the transcriptome of eutopic endometrium of adenomyosis with RNA‐Seq in Han Chinese population.

Gene expression profiles of endometrium from women with adenomyosis and matched controls had been explored with microarray platforms.^11^, ^20^ The study performed by Martinez‐Conejero et al^20^ was the first report to compare the transcriptome profiling of endometrium between adenomyosis and controls. All recipients enrolled in their study underwent oocyte donation during in vitro fertilization therapy, and the obtained endometrium was on LH + 7 days (window of implantation). Their results showed similar endometrial gene expression patterns between adenomyosis and controls with parametric test, and nonparametric method identified 34 differentially expressed genes. Considering the important role played by eutopic in the development of adenomyosis, the samples used by Herndon et al^11^ were eutopic endometrium in proliferative stage, which give clue to further studies. Limited by the research methods at that time, the microarray platform was with limited coverage; moreover, the enrolled patients with adenomyosis were no Asians. In our present study, we increased the sample size to compare the transcriptome profiling of endometrium in proliferative phase between adenomyosis and controls in Han Chinese population using RNA‐Seq in genome‐wide level. However, the dys‐regulated genes identified through the studies were rarely overlapped. The differences in adenomyosis diagnosis, sample collection stage and the testing platform might explain the different results as mentioned previously.^21^, ^22^

In this study, we demonstrated that the gene expression profile of the eutopic endometrium of adenomyosis was altered. The eutopic endometrium of adenomyosis and control samples could be clearly separated by PCA and hierarchical clustering. It was worth noting that the eutopic endometrium of adenomyosis was used in the RNA‐Seq. Indeed, a large number of genes (*CEBPB*, *SERPINE1*, *S100A9*, *CKS1B*, *CYR61*, *CD69*, *DUSP5*, *SOCS3*, *WEE1*, *DUSP1*, *EMP1*, *F3*, and *BTG1*) were significantly differentially expressed in both eutopic and ectopic endometrium, regardless of the proliferative phase and secretory phase. These findings further indicated that the aberrant gene expression occurred before the disease initiation and supported the hypothesis that adenomyosis might begin as an endometrial disease arising from aberrant gene expression in the endometrium before the establishment of lesions.

Adenomyosis is a complex disease with abnormal endometrial function. A previous study indicated the frequent coexistence of adenomyosis with endometrioid endometrial cancer.^23^ Eutopic endometrium of the women with adenomyosis showed abnormal biological processes, including decreased apoptosis, increased angiogenesis and proliferation, impaired cytokine expression and local production of oestrogens, which involved the pathogenesis of adenomyosis by enhancing the infiltration of the endometrium to the junctional zone myometrium and the growth of ectopic tissue.^24^ Consistent with previous findings, the significantly increased proliferation and decreased apoptosis were identified through the downstream analysis in our study. Besides, the pathway analysis in our study also indicated that IL‐6 signalling pathway and ERK/MAPK signalling pathway were significantly enriched. The IL‐6 signalling pathway was significantly increased in endometrial stromal cells after in vitro coculture with macrophage in adenomyosis which might play a role in the formation of ectopic tissues in adenomyosis.^25^ The ERK/MAPK signalling pathway was associated with the proliferation of uterine smooth muscle cells in the women with adenomyosis.^26^ The overlapping of our findings with the previous studies may reflect the universal mechanisms underlying the adenomyosis and the reliability of our data.

The identification of molecular differences within eutopic endometrium from women with adenomyosis is an important step in our understanding of the pathogenesis. The C/EBPβ, acting as a transcription factor regulating gene expression to control cellular proliferation, differentiation and metabolism,^27^ was identified as a core node in our network analysis based on the identified DEGs. C/EBPβ had been suggested as a critical regulator of human endometrial stromal proliferation and differentiation through cyclin E‐cdk2 and STAT3.^28^ However, its regulation role in adenomyosis had not been reported. In the study, we noticed that the mRNA and protein levels of C/EBPβ were both significantly increased in endometrium from women with adenomyosis regardless of proliferative and secretory phase.

It is well recognized that DNA methylation in promoters plays important roles in regulating gene expression and is a crucial contributor to the development of disease.^29^ In this study, *CEBPB* gene showed significant difference in DNA methylation level in the vicinity of TSS in endometrium between adenomyosis and matched controls, and 3 CpG units including CpG_1, CpG_15‐17 and CpG_30‐32 with the most significant difference were assumed to be the binding sites of transcription factors GATA‐1, MZF1 and GATA‐3. There are many studies reported that DNA methylation at specific CpG sites may affect the binding of transcription factors^30^, ^31^, ^32^; consequently, it is reasonable to speculate that the hypomethylation in the corresponding CpG sites has the potential to block transcription factor binding through interference with base recognition, thereby resulting in the altered C/EBPβ expression in endometrium from the patient with adenomyosis.

There are several limitations in our study. The first one is that the sample size is small and further study will be conducted with more samples. Another limitation is that we have not provided an experimental evidence to demonstrate the role of the genes. Thus, it is necessary to further explore the underlying roles of the identified pathways and molecules in adenomyosis.

In summary, we identified 373 DEGs and revealed key pathways and networks in the eutopic endometrium of adenomyosis patients. The aberrant methylation of the interesting gene candidate *CEBPB* might partially contribute to the deregulation of its expression. This study is the first to report the relationship between adenomyosis and the aberrant *CEBPB* expression and methylation and provides an important basis for further mechanistic studies on adenomyosis. We will further study the detailed role of *CEBPB* in the pathogenesis of adenomyosis.

## CONFLICT OF INTEREST

The authors confirm that there are no conflicts of interest.

## AUTHOR CONTRIBUTIONS

YX performed the experiments, analysed the data and drafted the manuscript. YS and BY collected clinical samples, performed the experiments and analysed the data. YY, YZ and TY participated in the collection clinical samples and conduction of the study. HH participated in the design of the study. JZ and HX designed this study, supervised the execution of this study and contributed to the drafting and revision of this manuscript.

## Data Availability

All data generated or analysed in this study are available upon request.
