# Supplementary material for: Transcriptome sequencing of adenomyosis eutopic endometrium: A new insight into its pathophysiology
Source: J Cell Mol Med. 2019 Oct 1;23(12):8381–91. doi: 10.1111/jcmm.14718 (PMC6850960; doi:10.1111/jcmm.14718)
Supplement: Supplementary file 1 [file JCMM-23-8381-s001.docx]

**Supplementary materials**

**Figures**

**
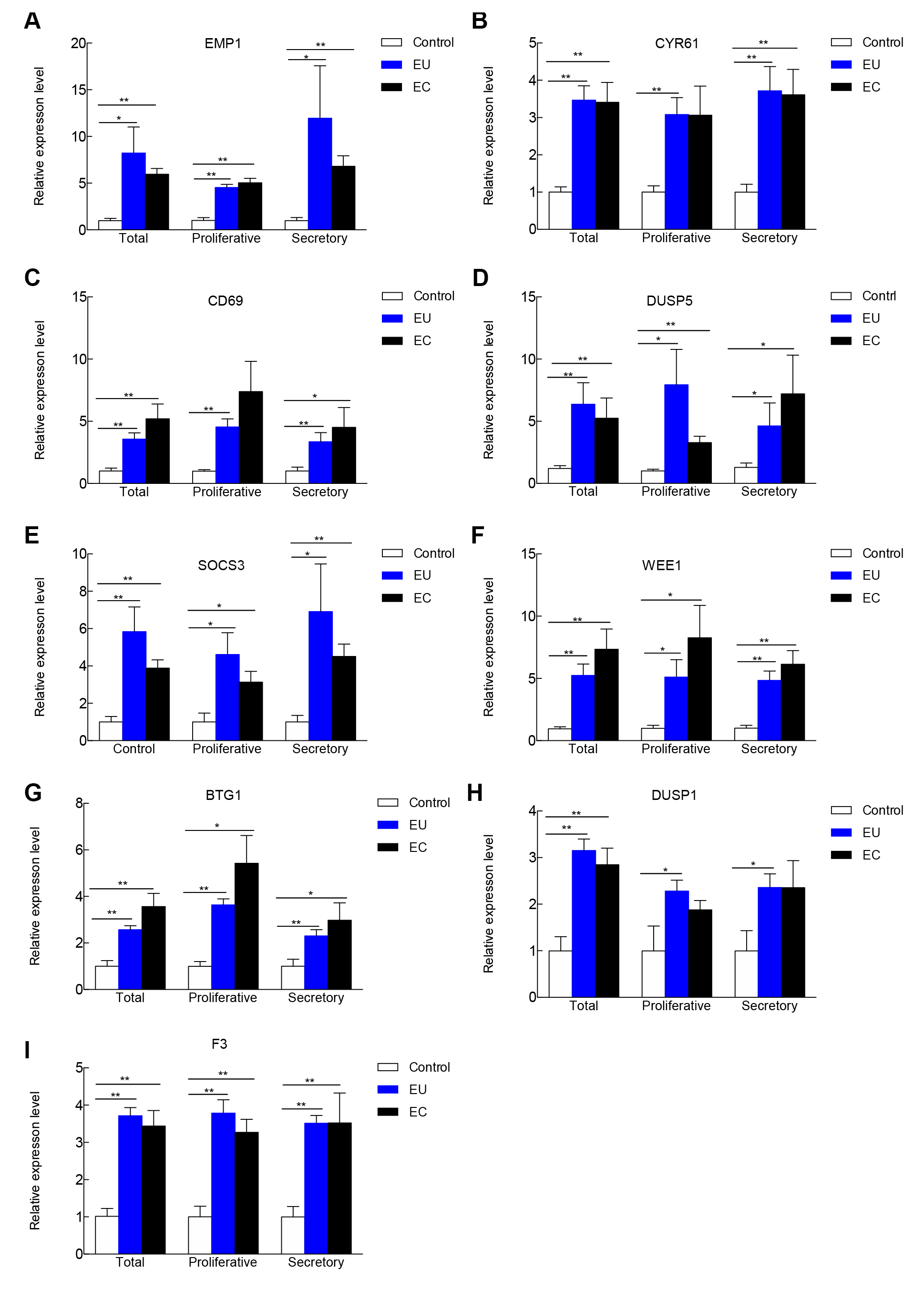
**

**Figure S1** **Validation of differently expressed genes by realtime PCR in eutopic, ectopic endometrium (N=14, 7 in proliferative stage and 7 in secretory stage) and matched controls (N=15, 5 in proliferative stage and 10 in secretory stage ). (A)** EMP1 **(B)** CYR61 **(C)** CD69 **(D)** DUSP5 **(E)** SOCS3 **(F)** WEE1 **(G)** BTG1 **(H)** DUSP1 **(I)** F3

**Tables**

**Table 1 The detailed clinical information of participants**

| **Serial Number** | **Age** | **Gravity and parity** | **Menstrual blood volume** | **Dysmenorrhea** | **Cycle phase histology** | **Clinical diagnosis** | **Experiments** |
| --- | --- | --- | --- | --- | --- | --- | --- |
| AN01 | 40 | G5P2 | Abound | Yes | Proliferative phase | Adenomyosis | RNA-Seq |
| AN02 | 50 | G1P1 | Abound | Yes | Proliferative phase | Adenomyosis | RNA-Seq |
| AN03 | 51 | G3P1 | Abound | Yes | Proliferative phase | Adenomyosis | RNA-Seq |
| AN04 | 48 | G2P1 | Abound | Yes | Proliferative phase | Adenomyosis | RNA-Seq |
| AN05 | 42 | G1P1 | Abound | Yes | Proliferative phase | Adenomyosis | RNA-Seq |
| AN06 | 47 | G4P1 | Abound | Yes | Proliferative phase | Adenomyosis | RNA-Seq |
| AN07 | 48 | G2P1 | Abound | Yes | Proliferative phase | Adenomyosis | qRT-PCR |
| AN08 | 47 | G3P2 | Abound | No | Secterotry phase | Adenomyosis | qRT-PCR |
| AN09 | 45 | G3P1 | Middle | Yes | Secterotry phase | Adenomyosis | qRT-PCR |
| AN10 | 37 | G3P1 | Abound | Yes | Secterotry phase | Adenomyosis | qRT-PCR |
| AN11 | 46 | G2P1 | Middle | No | Secterotry phase | Adenomyosis | qRT-PCR |
| AN12 | 49 | G2P1 | Middle | No | Proliferative phase | Adenomyosis | qRT-PCR |
| AN13 | 37 | G2P1 | Abound | Yes | Proliferative phase | Adenomyosis | qRT-PCR |
| AN14 | 50 | G1P1 | Middle | Yes | Proliferative phase | Adenomyosis | qRT-PCR |
| AN15 | 36 | G3P1 | Middle | No | Proliferative phase | Adenomyosis | qRT-PCR |
| AN16 | 33 | G1P1 | Middle | No | Secterotry phase | Adenomyosis | qRT-PCR |
| AN17 | 39 | G0P0 | Middle | Yes | Secterotry phase | Adenomyosis | qRT-PCR |
| AN18 | 46 | G3P1 | Abound | Yes | Secterotry phase | Adenomyosis | qRT-PCR |
| AN19 | 30 | G5P1 | Abound | Yes | Proliferative phase | Adenomyosis | qRT-PCR |
| AN20 | 46 | G2P1 | Middle | Yes | Proliferative phase | Adenomyosis | qRT-PCR |
| AN21 | 46 | G2P1 | Abound | Yes | Proliferative phase | Adenomyosis | HE |
| AN22 | 53 | G4P1 | Middle | Yes | Proliferative phase | Adenomyosis | HE |
| AN23 | 47 | G2P1 | Abound | Yes | Proliferative phase | Adenomyosis | HE |
| AN24 | 43 | G3P1 | Middle | Yes | Proliferative phase | Adenomyosis | HE |
| AN25 | 41 | G3P1 | Middle | Yes | Proliferative phase | Adenomyosis | HE |
| AN26 | 49 | G3P1 | Abound | Yes | Secterotry phase | Adenomyosis | HE |
| AN27 | 47 | G3P1 | Middle | Yes | Secterotry phase | Adenomyosis | HE |
| AN28 | 46 | G7P2 | Middle | Yes | Secterotry phase | Adenomyosis | HE |
| AN29 | 47 | G3P1 | Middle | Yes | Secterotry phase | Adenomyosis | HE |
| AN30 | 50 | G3P1 | Middle | Yes | Secterotry phase | Adenomyosis | HE |
| C01 | 47 | G4P2 | Middle | No | Proliferative phase | Myoma of uterus | RNA-Seq |
| C02 | 50 | G2P2 | Middle | No | Proliferative phase | Myoma of uterus | RNA-Seq |
| C03 | 50 | G2P1 | Middle | No | Proliferative phase | Myoma of uterus | RNA-Seq |
| C04 | 41 | G4P2 | Middle | No | Proliferative phase | Myoma of uterus | RNA-Seq |
| C05 | 43 | G4P1 | Middle | No | Proliferative phase | Myoma of uterus | RNA-Seq |
| C06 | 50 | G3P1 | Middle | No | Proliferative phase | Myoma of uterus | RNA-Seq |
| C07 | 47 | G2P2 | Middle | No | Proliferative phase | Myoma of uterus | qRT-PCR |
| C08 | 45 | G3P2 | Middle | No | Proliferative phase | CINIII | qRT-PCR |
| C09 | 54 | G3P1 | Middle | No | Proliferative phase | Myoma of uterus | qRT-PCR |
| C10 | 44 | G2P1 | Abound | No | Secterotry phase | Myoma of uterus | qRT-PCR |
| C11 | 43 | G4P3 | Middle | No | Secterotry phase | Myoma of uterus | qRT-PCR |
| C12 | 48 | G2P1 | Middle | Yes | Secterotry phase | Myoma of uterus | qRT-PCR |
| C13 | 42 | G2P1 | Abound | No | Secterotry phase | Myoma of uterus | qRT-PCR |
| C14 | 47 | G6P3 | Abound | No | Secterotry phase | Myoma of uterus | qRT-PCR |
| C15 | 53 | G1P1 | Middle | No | Secterotry phase | Myoma of uterus | qRT-PCR |
| C16 | 45 | G3P1 | Middle | No | Secterotry phase | Myoma of uterus | qRT-PCR |
| C17 | 54 | G2P1 | Abound | No | Secterotry phase | Myoma of uterus | qRT-PCR |
| C18 | 53 | G1P1 | Middle | No | Secterotry phase | Myoma of uterus | qRT-PCR |
| C19 | 30 | G2P1 | Middle | No | Proliferative phase | Myoma of uterus | qRT-PCR |
| C20 | 46 | G1P1 | Middle | No | Proliferative phase | Myoma of uterus | qRT-PCR |
| C21 | 48 | G3P1 | Middle | No | Secterotry phase | Myoma of uterus | qRT-PCR |
| C22 | 48 | G3P1 | Middle | No | Proliferative phase | Myoma of uterus | HE |
| C23 | 48 | G2P1 | Middle | No | Proliferative phase | Myoma of uterus | HE |
| C24 | 41 | G3P1 | Middle | No | Proliferative phase | Myoma of uterus | HE |
| C25 | 48 | G2P1 | Middle | No | Proliferative phase | Myoma of uterus | HE |
| C26 | 46 | G4P1 | Middle | No | Proliferative phase | Myoma of uterus | HE |
| C27 | 51 | G3P1 | Middle | No | Secterotry phase | Myoma of uterus | HE |
| C28 | 48 | G2P1 | Middle | No | Secterotry phase | Myoma of uterus | HE |
| C29 | 46 | G3P1 | Middle | No | Secterotry phase | Myoma of uterus | HE |
| C30 | 45 | G3P1 | Middle | No | Secterotry phase | Myoma of uterus | HE |
| C31 | 48 | G1P1 | Middle | No | Secterotry phase | Myoma of uterus | HE |

**Table S2 Sequences of PCR primers used in this study**

| Genes | Sequences (5'-3') |
| --- | --- |
| CEBPB-F | CAAAACTTTGGCACTGGGGC |
| CEBPB-R | CATGTGCGGTTGGTTTGGAC |
| SERPINE1-F | ATGCCCTCTACTTCAACGGC |
| SERPINE1-R | GGGCGTGGTGAACTCAGTAT |
| S100A9-F | CGGCTTTGACAGAGTGCAAG |
| S100A9-R | GCCCCAGCTTCACAGAGTAT |
| CKS1B-F | TATTCGGACAAATACGACGACG |
| CKS1B-R | CGCCAAGATTCCTCCATTCAGA |
| SPIN2B-F | CATGAAGACCCCCAACGCA |
| SPIN2B-R | AGAAATTCTGCAGCCCACGA |
| TCTN1-F | TGTTCAGTCCATCGTCATTCAG |
| TCTN1-R | GCAAAGGCTAAAGTGTCCAGC |
| CYR61-F | GCAAGGAGCTGGGATTCGAT |
| CYR61-R | TGCGAGGCTCCATTCCAAAA |
| CD69-F | AAGTTCCTGTCCTGTGTGCT |
| CD69-R | CTCTGGTAGCCAACCCAGTC |
| DUSP5-F | TCCTGAGTGTTGCGTGGATG |
| DUSP5-R | AGGATTTCAACTGGGCCACC |
| SOCS3-F | TCCAAACAGGGGACACTTCG |
| SOCS3-R | GGGGGTGTGACCATTTCCTT |
| WEE1-F | CTGAACAATGGGCCTCGTCT |
| WEE1-R | ATCCTATGGCTCGGGAGTGT |
| DUSP1-F | TTCCTGCAGTACCCCACTCT |
| DUSP1-R | TCCTCCACAGGGATGCTCTT |
| EMP1-F | TGTTGGTATTGCTGGCTGGT |
| EMP1-R | GCACTGTCTTGAGGGCATCT |
| BTG1-F | TCCAAGTTTCTCCGCACCAA |
| BTG1-R | GCATGGCTTTTCTGGGAACC |
| F3-F | CCTGGAGACAAACCTCGGAC |
| F3-R | CCCGGAGGCTTAGGAAAGTG |
| GAPDH-F | AGAAGGCTGGGGCTCATTTG |
| GAPGH-R | AGGGGCCATCCACAGTCTTC |
| MassAARRAY |  |
| CEBPB-MF | tag^a^- GTTGGAGGTTGTTTTGGAAGG |
| CEBPB-MR | T7^b^- CACCCAAAATCACACAATATAACAA |

^a^ tag, aggaagagag

^b^ T7, cagtaatacgactcactatagggagaaggct

**Table S3 Differentially expressed genes between adenomyosis and matched controls**

| **Symbol** | **Exp Log Ratio** | **Exp p-value** | **Exp False Discovery Rate (q-value)** |
| --- | --- | --- | --- |
| CPSF1 | -6.934 | 0.00005 | 0.0113 |
| LTF | -6.871 | 0.0005 | 0.0113 |
| MSRB3 | -6.121 | 0.00045 | 0.0113 |
| ELK2AP | -5.789 | 0.00005 | 0.0113 |
| TAC4 | -5.582 | 0.00105 | 0.0113 |
| MZB1 | -5.202 | 0.00095 | 0.0113 |
| IGLL1/IGLL5 | -4.56 | 0.00005 | 0.0113 |
| JCHAIN | -3.617 | 0.00005 | 0.0113 |
| S100A9 | -3.189 | 0.00005 | 0.0113 |
| SPIB | -2.766 | 0.00685 | 0.0349 |
| POU2AF1 | -2.481 | 0.0007 | 0.0113 |
| SHISA9 | -2.415 | 0.00005 | 0.0113 |
| S1PR4 | -2.244 | 0.0048 | 0.0248 |
| ITGB2 | -2.239 | 0.0011 | 0.0113 |
| LOC101929567 | -2.068 | 0.00605 | 0.0309 |
| GCHFR | -1.929 | 0.00005 | 0.0113 |
| LOC389033 | -1.916 | 0.00335 | 0.018 |
| TMEM160 | -1.9 | 0.00285 | 0.0158 |
| F12 | -1.892 | 0.004 | 0.0209 |
| CIB2 | -1.883 | 0.00115 | 0.0113 |
| GMFG | -1.881 | 0.0001 | 0.0113 |
| BIRC5 | -1.874 | 0.00005 | 0.0113 |
| ABCC9 | -1.861 | 0.0005 | 0.0113 |
| TMEM121 | -1.849 | 0.0003 | 0.0113 |
| VAV1 | -1.828 | 0.00565 | 0.029 |
| TNNC1 | -1.788 | 0.0001 | 0.0113 |
| CHCHD5 | -1.772 | 0.0004 | 0.0113 |
| SCNN1B | -1.73 | 0.00955 | 0.0475 |
| LINC00506 | -1.729 | 0.0052 | 0.0268 |
| GNLY | -1.718 | 0.00055 | 0.0113 |
| TMEM238 | -1.698 | 0.00065 | 0.0113 |
| PLAC9 | -1.667 | 0.0005 | 0.0113 |
| FUOM | -1.656 | 0.00645 | 0.0329 |
| CDCA5 | -1.637 | 0.0003 | 0.0113 |
| DIO2 | -1.637 | 0.0001 | 0.0113 |
| CYBB | -1.601 | 0.00445 | 0.0231 |
| SLC16A13 | -1.595 | 0.00695 | 0.0354 |
| AP2S1 | -1.589 | 0.00765 | 0.0387 |
| EXOSC4 | -1.579 | 0.00325 | 0.0175 |
| GJA4 | -1.566 | 0.0051 | 0.0262 |
| NDUFS6 | -1.553 | 0.00325 | 0.0175 |
| POC1A | -1.553 | 0.00505 | 0.026 |
| C1QTNF6 | -1.539 | 0.01 | 0.0496 |
| SDSL | -1.511 | 0.0051 | 0.0262 |
| MRPL27 | -1.493 | 0.00705 | 0.0358 |
| NUDT18 | -1.462 | 0.00785 | 0.0396 |
| NTHL1 | -1.453 | 0.00355 | 0.0188 |
| FBXW9 | -1.448 | 0.0058 | 0.0297 |
| CDT1 | -1.445 | 0.0085 | 0.0427 |
| SPC25 | -1.445 | 0.00855 | 0.0429 |
| ALKBH7 | -1.433 | 0.00995 | 0.0494 |
| CD14 | -1.412 | 0.00345 | 0.0183 |
| IFI27L2 | -1.4 | 0.00985 | 0.049 |
| VPS18 | -1.4 | 0.0008 | 0.0113 |
| FAM207A | -1.394 | 0.00715 | 0.0363 |
| PTGS1 | -1.394 | 0.0058 | 0.0297 |
| CORO1A | -1.392 | 0.00275 | 0.0153 |
| CDCA2 | -1.389 | 0.0062 | 0.0316 |
| E2F2 | -1.385 | 0.00485 | 0.025 |
| SAC3D1 | -1.384 | 0.00945 | 0.0471 |
| CKS1B | -1.383 | 0.00095 | 0.0113 |
| PMVK | -1.383 | 0.0016 | 0.0113 |
| MRPS34 | -1.38 | 0.0006 | 0.0113 |
| MFSD5 | -1.375 | 0.0005 | 0.0113 |
| CD7 | -1.373 | 0.00865 | 0.0434 |
| CDKN2AIPNL | -1.353 | 0.005 | 0.0258 |
| PEX11G | -1.348 | 0.0074 | 0.0375 |
| DPT | -1.343 | 0.00945 | 0.0471 |
| MRPL54 | -1.323 | 0.0046 | 0.0238 |
| OPHN1 | -1.316 | 0.00635 | 0.0324 |
| MRPL37 | -1.303 | 0.0012 | 0.0113 |
| NUDT1 | -1.294 | 0.0011 | 0.0113 |
| GPATCH3 | -1.29 | 0.0019 | 0.0118 |
| C1QA | -1.28 | 0.0073 | 0.037 |
| POLA2 | -1.277 | 0.00165 | 0.0114 |
| RPL39L | -1.273 | 0.00415 | 0.0217 |
| FCGR3A/FCGR3B | -1.27 | 0.004 | 0.0209 |
| ISOC2 | -1.267 | 0.00825 | 0.0416 |
| RNF187 | -1.26 | 0.0016 | 0.0113 |
| POLD2 | -1.247 | 0.0083 | 0.0418 |
| EIF4EBP1 | -1.246 | 0.00645 | 0.0329 |
| NDP | -1.219 | 0.0044 | 0.0228 |
| GPATCH4 | -1.209 | 0.00315 | 0.0171 |
| CLPP | -1.203 | 0.00315 | 0.0171 |
| COMTD1 | -1.201 | 0.0087 | 0.0436 |
| CDC20 | -1.2 | 0.0063 | 0.0321 |
| SF3B5 | -1.192 | 0.00245 | 0.0143 |
| NKG7 | -1.19 | 0.00245 | 0.0143 |
| ZNF775 | -1.19 | 0.00875 | 0.0438 |
| MFSD3 | -1.189 | 0.00275 | 0.0153 |
| GPAA1 | -1.181 | 0.00915 | 0.0457 |
| HN1 | -1.18 | 0.00335 | 0.018 |
| PLEKHJ1 | -1.18 | 0.0025 | 0.0143 |
| FEN1 | -1.174 | 0.00905 | 0.0453 |
| PYCARD | -1.17 | 0.0078 | 0.0394 |
| C1orf122 | -1.169 | 0.0041 | 0.0214 |
| DCTPP1 | -1.15 | 0.00615 | 0.0314 |
| ZNHIT2 | -1.149 | 0.00535 | 0.0275 |
| APEH | -1.146 | 0.008 | 0.0404 |
| CCNB2 | -1.136 | 0.0093 | 0.0464 |
| USP5 | -1.126 | 0.0087 | 0.0436 |
| ACP5 | -1.11 | 0.0072 | 0.0365 |
| TMEM37 | -1.107 | 0.0032 | 0.0173 |
| SDF2L1 | -1.103 | 0.00735 | 0.0373 |
| NDUFB7 | -1.099 | 0.00955 | 0.0475 |
| CCDC167 | -1.098 | 0.00695 | 0.0354 |
| RRM1 | -1.085 | 0.00585 | 0.0299 |
| S100A4 | -1.083 | 0.00505 | 0.026 |
| SDHAF1 | -1.081 | 0.0039 | 0.0204 |
| BORCS6 | -1.07 | 0.01 | 0.0496 |
| MRPL14 | -1.062 | 0.0097 | 0.0482 |
| VSTM4 | -1.055 | 0.00545 | 0.028 |
| CHMP6 | -1.044 | 0.009 | 0.045 |
| B3GALT6 | -1.003 | 0.00905 | 0.0453 |
| TIMM22 | -1.002 | 0.00785 | 0.0396 |
| IFNGR1 | 1.005 | 0.00595 | 0.0304 |
| RAB5A | 1.009 | 0.0084 | 0.0422 |
| DUSP6 | 1.022 | 0.0079 | 0.0399 |
| RHOB | 1.057 | 0.0092 | 0.046 |
| SEC31B | 1.063 | 0.00995 | 0.0494 |
| CLDN4 | 1.064 | 0.00405 | 0.0212 |
| R3HDM2 | 1.068 | 0.00955 | 0.0475 |
| SRF | 1.068 | 0.003 | 0.0164 |
| MESDC1 | 1.071 | 0.00805 | 0.0406 |
| RGL3 | 1.079 | 0.0086 | 0.0432 |
| RNF19A | 1.092 | 0.0093 | 0.0464 |
| TOM1L2 | 1.092 | 0.00445 | 0.0231 |
| PAN3 | 1.103 | 0.00935 | 0.0466 |
| PPP1R10 | 1.103 | 0.00245 | 0.0143 |
| LIMS3/LIMS4 | 1.106 | 0.01 | 0.0499 |
| NFKBID | 1.106 | 0.00725 | 0.0368 |
| MICAL3 | 1.115 | 0.00185 | 0.0116 |
| WWC3 | 1.128 | 0.0086 | 0.0432 |
| DENND4B | 1.131 | 0.0084 | 0.0422 |
| FAM189A2 | 1.134 | 0.0077 | 0.0389 |
| NFKB2 | 1.144 | 0.0097 | 0.0482 |
| SLC2A1 | 1.145 | 0.00355 | 0.0188 |
| NRBP2 | 1.149 | 0.00885 | 0.0443 |
| ULK3 | 1.151 | 0.00785 | 0.0396 |
| RRN3P1 | 1.155 | 0.0092 | 0.046 |
| USP54 | 1.157 | 0.0088 | 0.0441 |
| TP53INP2 | 1.164 | 0.00345 | 0.0183 |
| PXDC1 | 1.165 | 0.0052 | 0.0268 |
| ZSWIM6 | 1.166 | 0.00705 | 0.0358 |
| TGIF1 | 1.169 | 0.0039 | 0.0204 |
| KDM3A | 1.172 | 0.0037 | 0.0195 |
| HERC2P2 | 1.174 | 0.00475 | 0.0245 |
| LINC00174 | 1.175 | 0.0087 | 0.0436 |
| PDXDC2P | 1.178 | 0.00795 | 0.0401 |
| SLC38A2 | 1.183 | 0.00155 | 0.0113 |
| SHANK3 | 1.187 | 0.0056 | 0.0287 |
| TIPARP | 1.191 | 0.0085 | 0.0427 |
| SNX9 | 1.199 | 0.0089 | 0.0446 |
| CNKSR1 | 1.203 | 0.0066 | 0.0336 |
| NAMPT | 1.213 | 0.0073 | 0.037 |
| RALGDS | 1.213 | 0.003 | 0.0164 |
| TMCO4 | 1.216 | 0.0048 | 0.0248 |
| SS18L1 | 1.219 | 0.0059 | 0.0302 |
| CSF1 | 1.22 | 0.007 | 0.0356 |
| CLK3 | 1.223 | 0.0006 | 0.0113 |
| MIDN | 1.226 | 0.00445 | 0.0231 |
| mir-8 | 1.226 | 0.00785 | 0.0396 |
| DLC1 | 1.239 | 0.0007 | 0.0113 |
| CELSR1 | 1.243 | 0.0045 | 0.0233 |
| IER5 | 1.246 | 0.00465 | 0.0241 |
| POFUT2 | 1.259 | 0.00305 | 0.0167 |
| SUN1 | 1.264 | 0.00085 | 0.0113 |
| SEMA3B | 1.265 | 0.0091 | 0.0455 |
| ERF | 1.269 | 0.002 | 0.012 |
| CAPN15 | 1.27 | 0.0034 | 0.0183 |
| CHKA | 1.274 | 0.00995 | 0.0494 |
| TRIO | 1.275 | 0.00285 | 0.0158 |
| AGAP6 (includes others) | 1.288 | 0.00835 | 0.042 |
| BAG3 | 1.29 | 0.00105 | 0.0113 |
| FHL1 | 1.299 | 0.00705 | 0.0358 |
| MCL1 | 1.301 | 0.0086 | 0.0432 |
| AGER | 1.303 | 0.0021 | 0.0125 |
| MTMR11 | 1.309 | 0.00895 | 0.0448 |
| ZNF558 | 1.316 | 0.0044 | 0.0228 |
| KIAA1683 | 1.317 | 0.00475 | 0.0245 |
| AASS | 1.318 | 0.00155 | 0.0113 |
| CACNA1H | 1.321 | 0.0023 | 0.0134 |
| ZNF331 | 1.325 | 0.0048 | 0.0248 |
| PDGFB | 1.332 | 0.0037 | 0.0195 |
| ZFAND5 | 1.334 | 0.00115 | 0.0113 |
| ELMSAN1 | 1.336 | 0.00345 | 0.0183 |
| INPP5E | 1.337 | 0.0036 | 0.019 |
| VPS37B | 1.351 | 0.0065 | 0.0331 |
| IRS2 | 1.355 | 0.00925 | 0.0462 |
| WEE1 | 1.355 | 0.00075 | 0.0113 |
| IFRD1 | 1.356 | 0.001 | 0.0113 |
| SULT4A1 | 1.356 | 0.0083 | 0.0418 |
| ZNF83 | 1.359 | 0.00385 | 0.0202 |
| ITGA7 | 1.368 | 0.0095 | 0.0473 |
| KLF5 | 1.376 | 0.00095 | 0.0113 |
| KMT5C | 1.377 | 0.002 | 0.012 |
| TWIST1 | 1.377 | 0.0027 | 0.015 |
| PLA2G6 | 1.382 | 0.00435 | 0.0226 |
| CLK1 | 1.388 | 0.0003 | 0.0113 |
| RNF122 | 1.393 | 0.0014 | 0.0113 |
| LLGL2 | 1.408 | 0.00805 | 0.0406 |
| PPP1R15B | 1.425 | 0.0018 | 0.0116 |
| CEBPB | 1.426 | 0.00175 | 0.0116 |
| LOC729218 | 1.43 | 0.0022 | 0.0129 |
| ATXN7L2 | 1.433 | 0.00385 | 0.0202 |
| GABARAPL1 | 1.44 | 0.0004 | 0.0113 |
| RFX2 | 1.443 | 0.0018 | 0.0116 |
| SPRY4 | 1.448 | 0.00155 | 0.0113 |
| DLL1 | 1.454 | 0.00705 | 0.0358 |
| FRMD4B | 1.46 | 0.00175 | 0.0116 |
| TSC22D2 | 1.463 | 0.0013 | 0.0113 |
| VEGFA | 1.467 | 0.0002 | 0.0113 |
| ENO2 | 1.477 | 0.00525 | 0.027 |
| F3 | 1.479 | 0.00185 | 0.0116 |
| UGCG | 1.48 | 0.00035 | 0.0113 |
| JUN | 1.481 | 0.0074 | 0.0375 |
| LRP5L | 1.485 | 0.0074 | 0.0375 |
| ZFYVE28 | 1.485 | 0.00365 | 0.0193 |
| CELF6 | 1.486 | 0.00185 | 0.0116 |
| MZF1 | 1.488 | 0.00325 | 0.0175 |
| COL4A5 | 1.492 | 0.00665 | 0.0339 |
| ESM1 | 1.495 | 0.00275 | 0.0153 |
| KSR1 | 1.498 | 0.00025 | 0.0113 |
| MTRNR2L8 | 1.505 | 0.0036 | 0.019 |
| RGCC | 1.507 | 0.00185 | 0.0116 |
| CCNL1 | 1.508 | 0.00015 | 0.0113 |
| ARID5A | 1.511 | 0.0047 | 0.0243 |
| ITPRIP | 1.512 | 0.00035 | 0.0113 |
| HERC2P9 | 1.519 | 0.00425 | 0.0221 |
| TSPYL2 | 1.521 | 0.00245 | 0.0143 |
| GBP1 | 1.534 | 0.00055 | 0.0113 |
| PLIN5 | 1.544 | 0.003 | 0.0164 |
| FAM179A | 1.555 | 0.00975 | 0.0485 |
| EPHA2 | 1.561 | 0.0007 | 0.0113 |
| ABCG1 | 1.563 | 0.00235 | 0.0137 |
| NRP2 | 1.563 | 0.0041 | 0.0214 |
| SPRY1 | 1.564 | 0.0016 | 0.0113 |
| INSIG1 | 1.584 | 0.0001 | 0.0113 |
| EPOR | 1.588 | 0.0019 | 0.0118 |
| BCL3 | 1.591 | 0.0098 | 0.0487 |
| ETS2 | 1.598 | 0.00015 | 0.0113 |
| SERTAD1 | 1.602 | 0.0007 | 0.0113 |
| DNAJB4 | 1.604 | 0.00095 | 0.0113 |
| NFATC2 | 1.605 | 0.00995 | 0.0494 |
| CTGF | 1.614 | 0.00045 | 0.0113 |
| NCOA7 | 1.614 | 0.0011 | 0.0113 |
| KLF2 | 1.637 | 0.00325 | 0.0175 |
| EFNA1 | 1.64 | 0.0001 | 0.0113 |
| COL6A4P2 | 1.649 | 0.00595 | 0.0304 |
| RAP2C | 1.669 | 0.00365 | 0.0193 |
| VWA3A | 1.674 | 0.00055 | 0.0113 |
| SLC22A3 | 1.679 | 0.0026 | 0.0145 |
| ANXA1 | 1.681 | 0.00225 | 0.0132 |
| ADM | 1.682 | 0.00185 | 0.0116 |
| HBEGF | 1.682 | 0.0034 | 0.0183 |
| THBD | 1.704 | 0.00075 | 0.0113 |
| GADD45A | 1.706 | 0.00035 | 0.0113 |
| RGS1 | 1.707 | 0.0022 | 0.0129 |
| C2CD4B | 1.71 | 0.0004 | 0.0113 |
| THBS1 | 1.721 | 0.0078 | 0.0394 |
| GPRC5A | 1.724 | 0.00285 | 0.0158 |
| DGKD | 1.736 | 0.00175 | 0.0116 |
| CCDC40 | 1.738 | 0.0008 | 0.0113 |
| RND3 | 1.749 | 0.00005 | 0.0113 |
| BTG1 | 1.756 | 0.00385 | 0.0202 |
| PPP1R12B | 1.756 | 0.0023 | 0.0134 |
| CHRD | 1.775 | 0.00065 | 0.0113 |
| PFKFB3 | 1.786 | 0.00055 | 0.0113 |
| PLAUR | 1.796 | 0.00045 | 0.0113 |
| PIM1 | 1.802 | 0.00045 | 0.0113 |
| JUNB | 1.81 | 0.0012 | 0.0113 |
| NFATC1 | 1.83 | 0.0042 | 0.0219 |
| CACNA1C | 1.867 | 0.00175 | 0.0116 |
| ITPKC | 1.87 | 0.0002 | 0.0113 |
| MXD1 | 1.871 | 0.00045 | 0.0113 |
| PAK3 | 1.875 | 0.00925 | 0.0462 |
| RGS2 | 1.887 | 0.00085 | 0.0113 |
| CAPN6 | 1.903 | 0.00015 | 0.0113 |
| RGS16 | 1.905 | 0.0005 | 0.0113 |
| PER2 | 1.909 | 0.00005 | 0.0113 |
| DNAJB1 | 1.93 | 0.00595 | 0.0304 |
| MYADM | 1.938 | 0.00005 | 0.0113 |
| CD69 | 1.944 | 0.0025 | 0.0143 |
| C8orf4 | 1.945 | 0.00005 | 0.0113 |
| NFKBIA | 1.948 | 0.00465 | 0.0241 |
| LINC00893 | 1.96 | 0.00165 | 0.0114 |
| HPX | 1.963 | 0.00265 | 0.0148 |
| TMEM184A | 1.964 | 0.00025 | 0.0113 |
| NPIPB4 (includes others) | 1.992 | 0.00005 | 0.0113 |
| CYR61 | 2.003 | 0.00005 | 0.0113 |
| SLC2A3 | 2.004 | 0.00005 | 0.0113 |
| DUSP1 | 2.011 | 0.0025 | 0.0143 |
| GLIS1 | 2.025 | 0.0037 | 0.0195 |
| ELF3 | 2.032 | 0.00005 | 0.0113 |
| BCL6 | 2.033 | 0.00005 | 0.0113 |
| KLF4 | 2.033 | 0.00005 | 0.0113 |
| PTGIS | 2.049 | 0.00635 | 0.0324 |
| DUSP2 | 2.051 | 0.00005 | 0.0113 |
| BRINP1 | 2.052 | 0.0033 | 0.0178 |
| CD55 | 2.091 | 0.0001 | 0.0113 |
| CES4A | 2.093 | 0.00195 | 0.012 |
| LDLR | 2.112 | 0.00005 | 0.0113 |
| LOC102724428/SIK1 | 2.136 | 0.00005 | 0.0113 |
| ELN | 2.139 | 0.00725 | 0.0368 |
| LOC100133331 | 2.149 | 0.00095 | 0.0113 |
| ZC3H12A | 2.155 | 0.0002 | 0.0113 |
| GATA6 | 2.159 | 0.0018 | 0.0116 |
| LOC613037 | 2.162 | 0.00005 | 0.0113 |
| CD83 | 2.169 | 0.0002 | 0.0113 |
| SGK1 | 2.198 | 0.00005 | 0.0113 |
| ADAMTS8 | 2.221 | 0.00885 | 0.0443 |
| mir-132 | 2.221 | 0.00895 | 0.0448 |
| PMAIP1 | 2.224 | 0.00005 | 0.0113 |
| ERRFI1 | 2.227 | 0.00005 | 0.0113 |
| PIM3 | 2.254 | 0.00005 | 0.0113 |
| PPP1R15A | 2.264 | 0.0005 | 0.0113 |
| USP43 | 2.273 | 0.00005 | 0.0113 |
| KLF6 | 2.279 | 0.0001 | 0.0113 |
| CEBPD | 2.289 | 0.0002 | 0.0113 |
| IER3 | 2.297 | 0.00005 | 0.0113 |
| ADAMTS1 | 2.299 | 0.00005 | 0.0113 |
| OVOL1 | 2.308 | 0.0013 | 0.0113 |
| PLK3 | 2.312 | 0.0009 | 0.0113 |
| TNFRSF12A | 2.344 | 0.00005 | 0.0113 |
| mir-614 | 2.389 | 0.00005 | 0.0113 |
| BHLHE40 | 2.401 | 0.00005 | 0.0113 |
| NR4A2 | 2.404 | 0.0001 | 0.0113 |
| PHLDA2 | 2.424 | 0.00095 | 0.0113 |
| DUSP5 | 2.455 | 0.00005 | 0.0113 |
| LGI2 | 2.465 | 0.00085 | 0.0113 |
| EGR2 | 2.468 | 0.00005 | 0.0113 |
| CMYA5 | 2.501 | 0.003 | 0.0164 |
| CSRNP1 | 2.539 | 0.00005 | 0.0113 |
| EDN2 | 2.557 | 0.00005 | 0.0113 |
| DKK1 | 2.576 | 0.00025 | 0.0113 |
| HSPB7 | 2.603 | 0.00125 | 0.0113 |
| NR4A3 | 2.626 | 0.00005 | 0.0113 |
| POU5F1 | 2.641 | 0.00415 | 0.0217 |
| LIF | 2.656 | 0.00005 | 0.0113 |
| EMP1 | 2.657 | 0.00085 | 0.0113 |
| GADD45B | 2.66 | 0.00005 | 0.0113 |
| FOSB | 2.668 | 0.0004 | 0.0113 |
| RERG | 2.668 | 0.00565 | 0.029 |
| CNN1 | 2.674 | 0.0004 | 0.0113 |
| SOX9 | 2.694 | 0.00005 | 0.0113 |
| PER1 | 2.708 | 0.00005 | 0.0113 |
| PRELP | 2.721 | 0.00175 | 0.0116 |
| SCGB3A1 | 2.832 | 0.00005 | 0.0113 |
| TRIB1 | 2.93 | 0.00005 | 0.0113 |
| JPH2 | 2.977 | 0.0003 | 0.0113 |
| SOCS3 | 2.992 | 0.00005 | 0.0113 |
| TNFAIP3 | 2.996 | 0.00005 | 0.0113 |
| MAFF | 3.009 | 0.00005 | 0.0113 |
| ATF3 | 3.017 | 0.00005 | 0.0113 |
| C11orf96 | 3.028 | 0.00005 | 0.0113 |
| TNFAIP8L3 | 3.053 | 0.0002 | 0.0113 |
| ZFP36 | 3.057 | 0.00005 | 0.0113 |
| NR4A1 | 3.059 | 0.00005 | 0.0113 |
| EGR3 | 3.087 | 0.00005 | 0.0113 |
| FOSL1 | 3.091 | 0.0004 | 0.0113 |
| DUOXA1 | 3.109 | 0.0002 | 0.0113 |
| PDE4C | 3.151 | 0.00045 | 0.0113 |
| CXCL2 | 3.161 | 0.00005 | 0.0113 |
| NFASC | 3.319 | 0.00065 | 0.0113 |
| SERPINE1 | 3.392 | 0.00015 | 0.0113 |
| WHRN | 3.413 | 0.0022 | 0.0129 |
| PDLIM3 | 3.561 | 0.0007 | 0.0113 |
| CYP3A5 | 3.889 | 0.00385 | 0.0202 |
| GEM | 3.934 | 0.00005 | 0.0113 |
| IL1RL1 | 4.206 | 0.0035 | 0.0185 |
| ARC | 5.862 | 0.00005 | 0.0113 |
| SELE | 6.597 | 0.00565 | 0.029 |
| REXO1 | 11.127 | 0.00005 | 0.0113 |
|  |  |  |  |
